# Acute successful management of a chainsaw injury to the roof of the left chest

**DOI:** 10.1308/rcsann.2022.0170

**Published:** 2023-05-24

**Authors:** E Umotong, E Quinn, S Qadri, R Chandran, J Zacharias

**Affiliations:** ^1^ Blackpool Victoria Hospital, UK; ^2^ Salford Royal Foundation Trust, UK

**Keywords:** Chainsaw, Left chest, Trauma

## Abstract

This case report chronicles the experience of prompt resuscitation, beginning with the patient, and immediate surgery following the fatal malfunction of a chainsaw. The injuries were atypical for chainsaw injuries and involved total transection of the left subclavian artery and vein, total transection of the left brachial plexus and laceration to the apex of the left lung, among other injuries. A coordinated effort allowed for successful repair of the life- and limb-threatening injuries so that the patient could return to his young family in time for his 40^th^ birthday.

## Background

This case report presents the history of a 39-year-old male who sustained life- and limb-threatening injuries to the roof of his left thorax following the malfunction of a chainsaw. Though chainsaw accidents have been reported previously in the literature, none reported involve successful management of injuries to the roof of the thorax.

## Case history

A 39-year-old man with no previous medical conditions and an allergy to penicillin was cutting wood with a chainsaw when he lost control of the device after his T-shirt became entangled in the blades of the chain. He reported directing the blade away from his face which inadvertently caused the blade to make contact with his left upper chest. Once freed from the chainsaw, he placed himself on the floor in a supine position, applied direct compressive pressure to the exsanguinating wound and called for his wife who was present in a neighbouring room. His wife attended immediately and called for an ambulance before, under instruction of the patient, she substituted the patient’s hand and continued with direct compression of the haemorrhaging wound. The helicopter emergency medical service presented within 13 minutes of receiving the emergency call.

On arrival, the paramedic team found the patient lying supine with a Glasgow Coma Scale (GCS) score of 13 (E3V4M6). He was pale in complexion and there was large laceration from the left clavicle to beneath the left pectoral muscle with fatty tissue, bone and lung tissue exposed and with evidence of an air leak through the wound. There was catastrophic haemorrhage from the left clavicle and chest and the patient was hypotensive. They also noted neurovascular compromise to the left upper limb with no muscle tone or sensation in the left arm and absent pulses. Observations revealed oxygen saturation of 74% on 15l oxygen and blood pressure reading of 70/19.

At the scene, the patient’s open wound was packed with 4–5 rolls of Celox and direct pressure was applied. The decision was made to transfer the patient to a facility with cardiothoracic services due to the presence of an air leak. He was transported by ground escort (high risk of requiring thoracotomy made aircraft transport inappropriate) and they left the scene 46 minutes after arrival. The patient and his wife commented on how calm and coordinated they found the paramedic staff’s out-of-hospital, advanced trauma emergency care.

En route to the tertiary centre, four intravenous lines were sited and he received 1g tranexamic acid, morphine analgesia and eight units of blood products (four RBC, four prethawed plasma). The patient had developed a left-sided tension haemothorax for which intrapleural catheterisation (28Fr drain, 5^th^ intercostal space) was performed under 50mg ketamine sedation; 2.5l of frank blood drained through the chest drain. He received further smaller doses of ketamine (total 100mg, 50/20/30) and midazolam (total 3mg) and a transfusion of calcium chloride.

On arrival at hospital, his GCS was 10 (E3V2M5) and blood pressure was 60/21. His left arm was grossly oedematous with reduced tone, power 0/5 and absent sensation. Due to the severity of his chest wounds, he was transferred straight to the operating theatre. 

**Figure 1:**
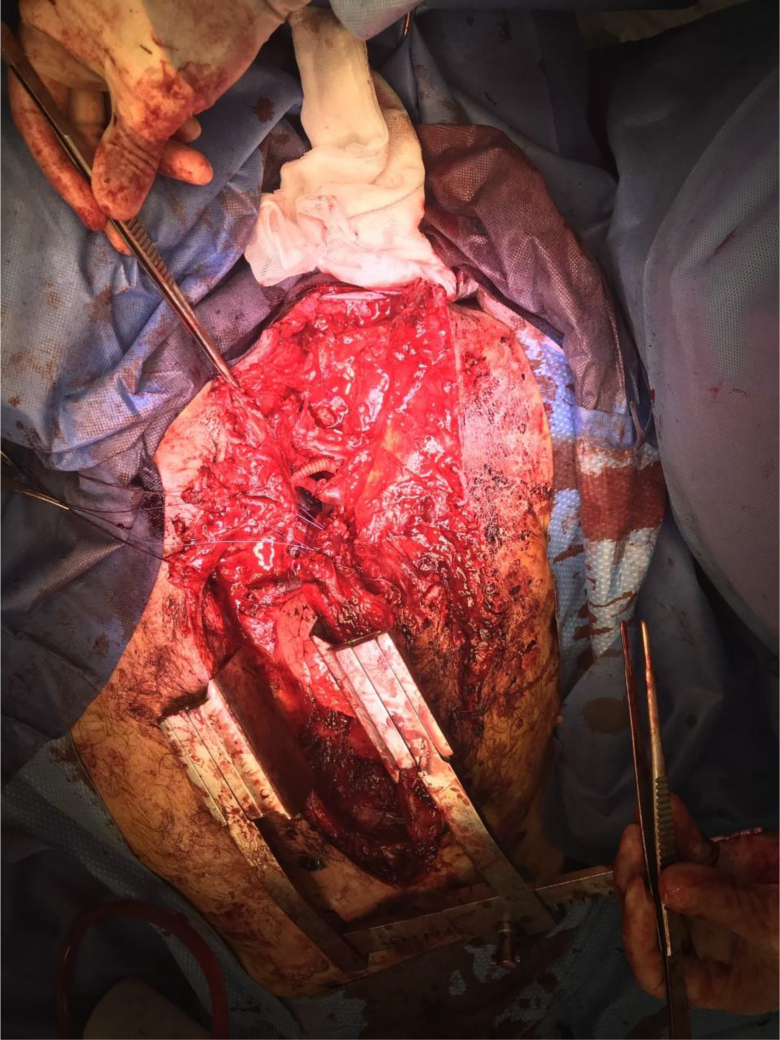
Intra-operative mages of the patient’s chest following sternotomy. The oblique chainsaw wound with ragged edges can be seen to the left of the sternotomy wound extending to the distal clavicle. At this point, the subclavian artery and vein have been grafted and the ends of the brachial plexus (transected at the level of the cords) were being identified.

Surgical exploration identified a deep, oblique Y-shaped 12cm cut to the upper left chest with ragged edges (Figure 1). The tail was towards the acromial end of the clavicle and the heads were towards the manubrium joint and 2–3cm below this, just lateral to the midline. Through this deep cut, skin, subcutaneous fat, pectoralis major and minor, and intercostal muscles were dissected and exposed. In addition, the first rib distally and clavicle were completely fractured. Beneath these, the brachial plexus was transected at the level of the cords and the subclavian artery and vein were also completed dissected and heavily retracted due to vasospasm. Lacerations to the apex of the upper lobe of the left lung were also found. The phrenic nerve was identified crossing the field and was preserved.

**Figure 2:**
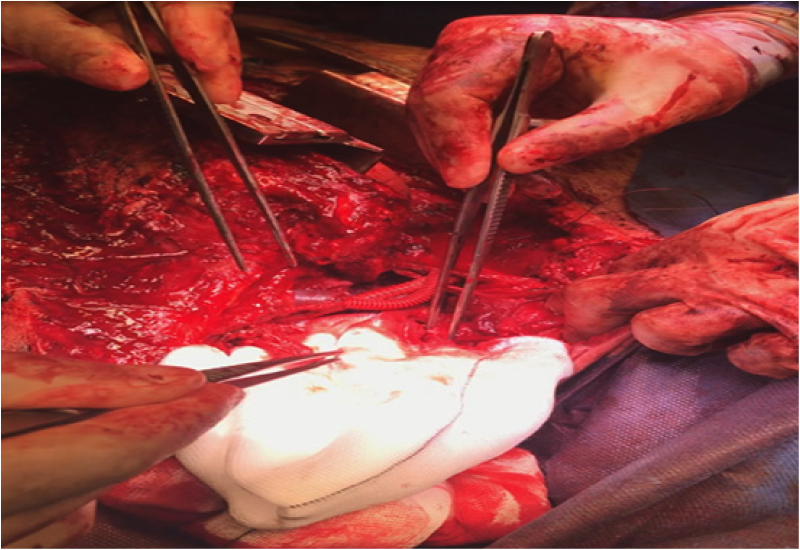
Identification of the ends of the brachial plexus following graft repair of the subclavian vessels

**Figure 3:**
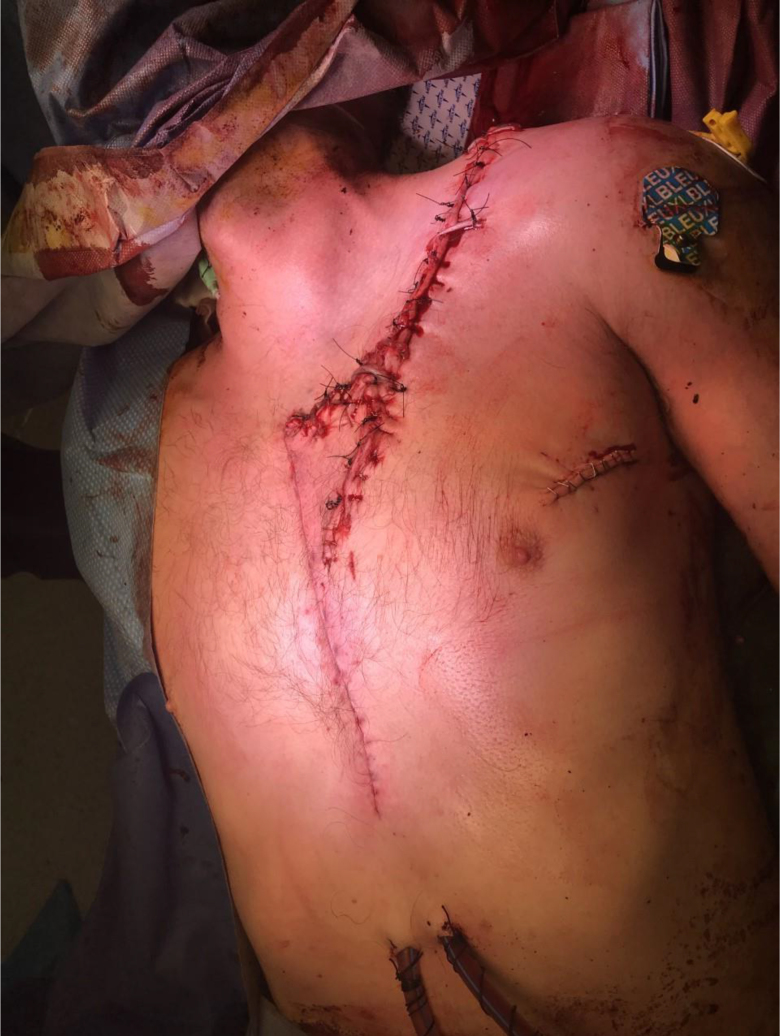
Use of deep tension sutures to close the defects primarily at the end of the operation

Sternotomy was performed and the edges of the subclavian vessels were identified and an interposition graft using 6mm Dacron was fashioned. The edges of the brachial plexus were also identified (Figure 2) and fixed with vicryl sutures. The lung lacerations were repaired with primary suturing techniques before closure and hence we were able to conserve the upper lobe. The edges of the wound were freshened and deep tension sutures were used to close the defect primarily (Figure 3).

Postoperatively, the patient was transferred to the cardiac intensive care unit. He began reporting paraesthesia to the left upper limb, though the limb remained grossly oedematous with absent power, sensation and reflexes. He developed a chyle leak on postoperative day 3 once he started oral feeds, for which he received octreotide and a fat-free diet. The chest drainage settled down very quickly and he recovered from this injury with conservative management. Postoperative images revealed occlusion to the reconstructed left subclavian vein for which therapeutic dalteparin was initiated. He received a total of 24 units of RBC, 4 units FFP and 2 of platelets and spent 8 days in the intensive care unit.

Upon discharge, he was transferred for specialist brachial plexus repair and underwent cable grafting of the left brachial plexus and refashioning of the subclavian artery graft 21 days following the initial trauma.

He was seen in clinic 32 days following the injury. Despite lacking power 0/5 in all myotomes in the left upper limb, the limb was vascularly intact, sensation improving and he was clinically well. His chest x-ray showed clear lungs with no evidence of phrenic damage.

## Discussion

Chainsaw accidents are a rare cause of penetrating chest trauma. In a review of 330 chainsaw injury patients, Haynes *et al* found that 48 patients required hospital admission and, of these, only 2 injuries affected the chest and none were to the roof of the thorax.^1^ There was a typical pattern of injury; with the majority occurring on the left and jagged wound edges being a common feature. It was found that contact with bone abruptly slowed the revolving chain and thus limited further damage, as was the case in the history presented.

Truncal vascular injuries are known to carry high mortality due to blood loss, which is difficult to access and control with direct compression. In this case, the patient and his wife, under the patient’s direction, were able to limit blood loss and prolong survival through their direct compression. The patient reported that the bleeding resumed when the paramedic team took over and he provided direction to help stem the blood loss once again. Thus, it would seem that there is an element of proprioceptive feedback required for complete haemorrhagic control when applying direct compressive pressure to truncal vascular injuries that is yet to be explored and discussed in the literature.

Celox is a nontoxic, biodegradable, chitin-derivative (poly-N-acetyl glucosamine) haemostatic agent that is available in powder, gauze and nasal tampon forms for ease of application. When it contacts blood, it encases red blood cells and platelets as it swells, gels and sticks together, forming a gel-like plug at the local site to promote clot formation and provide a barrier against ongoing haemorrhage. Animal studies have found it also increases tissue factor (TF) activities, with TF going on to initiate the coagulation cascade.^2^ Through this TF-mediated thrombin-burst, potent vasospasm can be induced through activation of cell signaling pathways,^3–6^ and the authors suggest that this contributed to the life-saving haemostatic properties of Celox that were demonstrated in this case, although the vasospasm could also have been impacted by additional factors such as exposure to cold and/or dryness.

In the patient with penetrating injuries, and where there is doubt, mandatory surgical exploration has been advocated as the history, clinical examination and imaging may not accurately reflect the extent of injury. While hospital stay is prolonged in patients who have undergone mandatory surgery and negative exploration (4.2 vs 2.8 days for those managed selectively and do not require surgery), the overall costs are similar and significant morbidity is rarely attributable to a negative surgical exploration.^7–10^ It is important to consider that lesions may still be missed during surgical exploration,^11^ and units with experienced angiographers and medical and nursing staff suitably trained for frequent repeated examinations may safely manage patients more selectively.^12^

The availability of a cardiothoracic surgeon at the unit helped manage the vascular and lung injuries in a timely fashion by a single surgeon. There was the option of tying off the lacerated vessels, but an attempt was made to both save life and limb in view of the relative youth of the patient. The team were aware of the high likelihood of occlusion of the subclavian vein reconstruction but it helped reduce the oedema in the upper limb in the immediate postoperative care while collateral channels developed around the axillary and clavicular circulation.

Due to the fragility of the thoracic duct and variability in anatomy, iatrogenic thoracic duct injury causing chyle leak may complicate various neck and thoracic surgeries. Fortunately, successful surgical outcome can nonetheless be achieved with prompt diagnosis and treatment. If intraoperative ligation of the thoracic duct is not possible, conservative measures include bedrest to reduce chyle flow through physical activity, stool softeners to reduce intrathoracic and intrabdominal pressures with bowel movements, close monitoring for dehydration and malnutrition due to high-volume fluid shifts with protein and electrolyte losses (with or without intravenous fluids to maintain euvolaemia and electrolyte replacements) and a nonfat, low-fat or medium chain fatty acid diet is crucial to reduce the availability of substrates for chyle production. Medical management of chyle leaks include orlistat, which prevents lipid absorption and assists in reducing chyle formation; somatostatin, to reduce chyle production; or octreotide, somatostatin’s longer-acting counterpart, to reduce chyle formation. Surgical exploration is advised if significant reductions in drain output are not seen after 5 days with conservative measures.^13–18^

## Conclusion

This case highlights the importance of a well-coordinated multidisciplinary approach, beginning with the patient, and signals the benefits of widespread patient education on initial trauma management.

Due to the commercial availability of chainsaws, it is likely that the emergency theatre will continue to host chainsaw accidents; however, ongoing reports of the dangers of this power tool may help place pressure on the industry to enforce mandatory safety precautions on the use of these devices.

## Conflict of interest

J. Zacharias receives consulting and speaker fees from Edward Lifesciences, Medtronic, Terumo and Cambridge Medical Robotics.
